# A Rare Case of Cedecea Davisae Bacteremia Presenting as Biliary Sepsis

**DOI:** 10.7759/cureus.5298

**Published:** 2019-08-01

**Authors:** Venkata Sowjanya Kanakadandi, Manbeer S Sarao, Jessica M Cunningham

**Affiliations:** 1 Internal Medicine, University of Pittsburgh Medical Center Pinnacle, Harrisburg, USA; 2 Internal Medicine, Griffin Hospital, Derby, USA

**Keywords:** cedecea davisae, biliary sepsis, immunocompromised, gram-negative

## Abstract

Cedecea davisae is a gram-negative, non-sporulating motile rod-shaped bacteria of the Enterobacteriaceae family. It is an opportunistic pathogen in advanced-aged patients with many comorbid diseases and the immunosuppressed. To the best of our knowledge, only 12 cases of C. davisae bacteremia have been reported in the literature. Here we discuss the 13th case of C. davisae bacteremia, which is the first reported case presenting as biliary sepsis.

A 41-year-old female, on prednisone for minimal change disease, presented with nausea, vomiting, fever, and diarrhea. She had dry mucous membranes, scleral icterus, and elevated liver enzymes. Blood cultures revealed Cedecea davisae. She improved after management with broad-spectrum antibiotics.

Further studies are needed to understand its role in the mode of transmission, the spectrum of infection, and treatment options. There is a need for physicians to be cognizant of emerging pathogens and address their antibiotic resistance profiles.

## Introduction

*Cedecea davisae* (*C. davisae*), a member of the Enterobacteriaceae family, is a gram-negative, oxidase negative, lipase positive, non-sporulating motile rod-shaped bacteria [1]. It has been isolated from various clinical specimens including sputum (the most common source), urine, cutaneous and oral ulcers, scrotal abscesses, peritoneal dialysis fluid, and the gallbladder. It is an opportunistic pathogen in advanced-aged patients with many comorbid diseases and the immunosuppressed. The combination of AmpC production and porin deficiency in the cell wall contributes to its multidrug resistance [2]. It possesses plasmid carrying multidrug resistance genes, through which it can transfer its genetic materials to other susceptible bacteria in the same ecosystem [3]. To the best of our knowledge, only 12 cases of *C. davisae* bacteremia have been reported in the literature. Here we discuss the 13th case of *C. davisae* bacteremia, which is the first reported case presenting as biliary sepsis.

## Case presentation

A 41-year-old female, with a past medical history of minimal change disease, diagnosed two years ago, being treated with a tapering dose of prednisone (on 7.5 mg daily at the time of admission) and biliary cancer status post percutaneous cholecystostomy drain placed 10 days prior, presented to the emergency department with nausea, vomiting, fever, and diarrhea of one-day duration. This was associated with a decreased output from the cholecystostomy drain.

Six weeks prior to this presentation, the patient had routine monthly blood work done by her nephrologist and was noted to have bilirubin in her urine. Her liver function tests were then ordered which were abnormal and a right upper quadrant ultrasound showed moderate to marked diffuse intrahepatic bile duct dilation with a suspected obstructing mass at the porta hepatis. Outpatient work-up including alpha-1 antitrypsin, ceruloplasmin, antimitochondrial antibody, Hepatitis B, Hepatitis C, cytomegalovirus, iron, and ferritin were all negative or within the normal range. Her antinuclear antibody titer was at 1:40. Given concern for an obstructing mass, the patient was referred to the emergency room for expedited endoscopic retrograde cholangiopancreatography (ERCP). A computed tomography (CT) of the abdomen was done and it confirmed a mass at the hepatic hilum with associated biliary obstruction.

The patient had ERCP where she was found to have a high-grade stricture at the hepatic bifurcation. A sample was collected and sent for cytology and a stent was placed within the left intrahepatic biliary tree. The cytology from the ERCP returned as rare atypical cells with no malignant cells. A follow-up magnetic resonance imaging (MRI) of the abdomen was ordered and again showed a solid mass at the porta hepatis. A CT-guided liver biopsy was performed and pathology was pending when the patient was referred to interventional radiology due to continued significant hyperbilirubinemia. The patient underwent a cholangiogram and after some difficulty passing a wire, an internal/external biliary drainage catheter was placed. The drain was putting out 500-600 ml per day of bile.

She was undergoing prep for colonoscopy to rule out a colonic source of malignancy when her biliary output began to decrease and she developed the symptoms which prompted her return to the emergency room.

In the emergency room, she appeared to be in distress, with a temperature of 99.3ºF, heart rate 142/min, blood pressure 92/52 mmHg, respiratory rate 18/min and SpO2 98% on room air. Her physical exam was significant for dry mucous membranes, scleral icterus, and a non-tender abdomen. Her laboratory investigations revealed sodium 133 mmol/L, alkaline phosphatase 486 U/L, aspartate transaminase 122 U/L, alanine transaminase 212 U/L, bilirubin 14.6 mg/dL (direct bilirubin 8.0 mg/dL), and international normalized ratio 1.4. Management, according to the sepsis protocol, included intravenous (IV) fluids and the broad-spectrum antibiotics cefepime and vancomycin. A contrast-enhanced CT of the abdomen confirmed good biliary drain position. It also revealed vicarious excretion of bile and an enlarged periportal lymph node. Metronidazole was added to the treatment regimen. The patient received aggressive fluid resuscitation in the intensive care unit, with a total of 8.5 L of IV fluid being given over 30 hours. Initial blood cultures grew gram-negative rods, so Vancomycin was discontinued and infectious disease was consulted. Eventually, blood cultures revealed *Cedecea davisae* identified on micro scan and crystal identification, resistant to ampicillin, ceftriaxone, and cefuroxime. Repeat blood cultures were negative at 48 hours. Infectious disease recommended transitioning to Ciprofloxacin 500 mg orally every 12 hours and Metronidazole 500 mg orally every 8 hours to complete a 14-day course. At the time of discharge, she was feeling well and her vital signs were all within normal limits.

## Discussion

The genus Cedecea is a member of the family Enterobacteriaceae. Its name comes from the abbreviation “CDC,” for the Centers for Disease Control and Prevention (CDC), where the initial group of isolates, “Enteric Group 15,” were discovered [4]. The genus Cedecea consists of six species. Three of these, *C. davisae*, *Cedecea lapagei*, and *Cedecea neteri* (formerly known as Cedecea species 4 or Cedecea species 002​​​​​​) have been fully identified and named after the American bacteriologist Dr. Betty R. Davis, the British bacteriologist Dr. Stephen P. Lapage and the American physician-microbiologist Dr. Erwin Neter respectively, who all have made significant contributions to the Enterobacteriaceae family [5]. The remaining three unnamed species are Cedecea species 001 (also known as Cedecea species 3), Cedecea species 012 (also known as Cedecea species 5), and Cedecea species 6.

The species was first identified in 1977 by a group of microbiologists who classified it under the genus of Enterobacteriaceae. The organism was found to be lipase positive, DNase and gelatin negative with resistance to colistin and cephalothin [6]. In 1981, Cedecea was designated as a separate genus of the Enterobacteriaceae family as it was phenotypically distinct from the other members of the family and given its name.

There have been very few cases of reported Cedecea infections and only 12 *C. davisae* infections (see table) since its discovery in 1977. Sputum, gall bladder, skin wounds, and abscesses are some of the sources the species has been isolated from. However, the natural habitat of the organism is yet to be determined. Infections such as pneumonia, urinary tract infection, and sepsis, that have been documented in the past have been rare and were largely caused by the lapagei and neteri species. *Cedecea davisae* has been isolated in reported cases of bacteremia, which also have been very few.

One of the first known cases of *C. davisae* bacteremia was reported by Perkins et al. in 1986, who isolated the organism from sputum, central venous and Swan-Ganz catheters, along with *Staphylococcus epidermidis*. The patient did have sputum positivity, but no pneumonia; hence, there was a consideration given that the infection was acquired from the patient’s own flora. In most cases, patients that did have infections with Cedecea were found to be immunocompromised with uncontrolled diabetes mellitus, renal transplant, chronic kidney stage V, chronic bronchitis or having an underlying malignancy. Based on previously reported cases, there also appears to be an association with the presence of catheters, from which the organism has been isolated. Our current knowledge on this organism has only been from the few previously reported cases, which indicate an opportunistic nature to the infections caused by it. Further studies are needed to fully explore the pathogenicity of this organism.

Though infections with *C. davisae* or the genus Cedecea in itself are rare, treating them is a challenge due to their broad-spectrum antibiotic resistance. Apart from resistance to colistin and cephalothin, previously described cases reported also report microbial resistance to, cefuroxime, ceftazidime, ampicillin, tetracycline, cefoxitin, piperacillin, nitrofurantoin, ciprofloxacin, several aminoglycosides, and third-generation cephalosporins. The combination of AmpC production and porin deficiency in the cell wall contributes to the multidrug resistance.

Our case describes the 13th case of C. davisae bacteremia and adds to the limited literature available. To the best of our knowledge, this is the first case of *C. davisae* bacteremia presenting as biliary sepsis. As seen in Table 1, the infection could have various presentations.

**Table 1:** Cases of Cedecea davisae in Literature DM: diabetes Mellitus; CHF: congestive heart failure; HTN: hypertension; COPD: chronic obstructive pulmonary disease; AML: acute myeloid leukemia; CF: cystic fibrosis; CKD: chronic kidney disease; UTI: urinary tract infection

Year	Age	Gender	Comorbidities	Site
1981 [7]	65	F	DM, CHF, HTN	Sputum
	76	M	HTN, arteriosclerotic heart disease	Sputum
1983 [8]	50	M	HTN, CHF, alcoholic hepatitis	Scrotal abscess
1986 [6]	70	F	Heart disease, bronchitis, COPD	Blood
2008 [4]	67	M	DM	Blood, Leg ulcer
2010 [9]	42	M	s/p Renal transplantation, DM, HTN	Oral ulcer
2011 [10]	52	M	AML, Clostridium difficile colitis	Blood
2012 [11-12]	54	M	Sigmoid colon cancer	Blood
	20	F	CF, DM	Sputum
2013 [1]	77	F	DM, HTN, CKD	Blood and permacath catheter tip
2015 [13]	57	F	Atrophic rhinitis with mucocele	Mucocele
2018 [5]	76	M	UTI s/p prostatectomy	Urine

## Conclusions

As the species has an inherent resistance to a broad spectrum of antibiotics, management is challenging, particularly in immunocompromised individuals. Hence, further studies are needed to understand *C. davisae’s* role in the mode of transmission, the spectrum of infection and treatment options.
